# A Sorghum MYB Transcription Factor Induces 3-Deoxyanthocyanidins and Enhances Resistance against Leaf Blights in Maize

**DOI:** 10.3390/molecules20022388

**Published:** 2015-01-30

**Authors:** Farag Ibraheem, Iffa Gaffoor, Qixian Tan, Chi-Ren Shyu, Surinder Chopra

**Affiliations:** 1 Department of Plant Science, The Pennsylvania State University, University Park, PA 16802, USA; E-Mails: farag100@mans.edu.eg (F.I.); sig2@psu.edu (I.G.); qzt101@psu.edu (Q.T.); 2 Intercollege Graduate Degree Program in Plant Biology, Pennsylvania State University, University Park, PA 16802, USA; 3 Botany Department, Faculty of Science, Mansoura University, AlMansoura, 35516, Egypt; 4 MU Informatics Institute, University of Missouri, Columbia, MO 65201, USA; E-Mail: mailto:shyuc@missouri.edu

**Keywords:** anthracnose, 3-deoxyanthocyanidins, *Colletotrichum*, flavan-4-ols, transgenic maize, *yellow seed1*

## Abstract

Sorghum responds to the ingress of the fungal pathogen *Colletotrichum sublineolum* through the biosynthesis of 3-deoxyanthocyanidin phytoalexins at the site of primary infection. Biosynthesis of 3-deoxyanthocyanidins in sorghum requires a MYB transcription factor encoded by *yellow seed1* (*y1*), an orthologue of the maize gene *pericarp color1* (*p1*). Maize lines with a functional *p1* and flavonoid structural genes do not produce foliar 3-deoxyanthocyanidins in response to fungal ingress. To perform a comparative metabolic analysis of sorghum and maize 3-deoxyanthocyanidin biosynthetic pathways, we developed transgenic maize lines expressing the sorghum *y1* gene. In maize, the *y1* transgene phenocopied *p1-*regulated pigment accumulation in the pericarp and cob glumes. LC-MS profiling of fungus-challenged *Y1-*maize leaves showed induction of 3-deoxyanthocyanidins, specifically luteolinidin. *Y1-*maize plants also induced constitutive and higher levels of flavonoids in leaves. In response to *Colletotrichum graminicola*, *Y1-*maize showed a resistance response.

## 1. Introduction

Maize (*Zea mays* L.) is an important cereal crop. In 2013, the total area planted under maize for all purposes in the United States amounted to 95.37 million acres, with about 87.67 million acres for grain production (U.S. Department of Agriculture, National Agricultural Statistics Service). In the field, maize plants frequently encounter a wide variety of pathogens. Anthracnose caused by *Colletotrichum graminicola* (Ces.) G. W. Wils. and southern corn leaf blight caused by *Cochliobolus heterostrophus* (Drechsler) are among the most serious fungal diseases that affect productivity.

Application of synthetic fungicides is among the strategies used to control fungal infections, but their cost and environmental impact are a concern for producers and consumers. To prevent further epidemics and reduce the need for synthetic chemicals, there is an ongoing search for crop germplasm with natural resistance [1]. Metabolic engineering of defense-related compounds has proven effective in enhancing plant performance against biotic stress [2,3]. This approach offers an opportunity to either transfer a complete defense-related metabolic pathway or activate a preexisting one by the transfer of genes between distant plant species [4,5].

**Figure 1:**
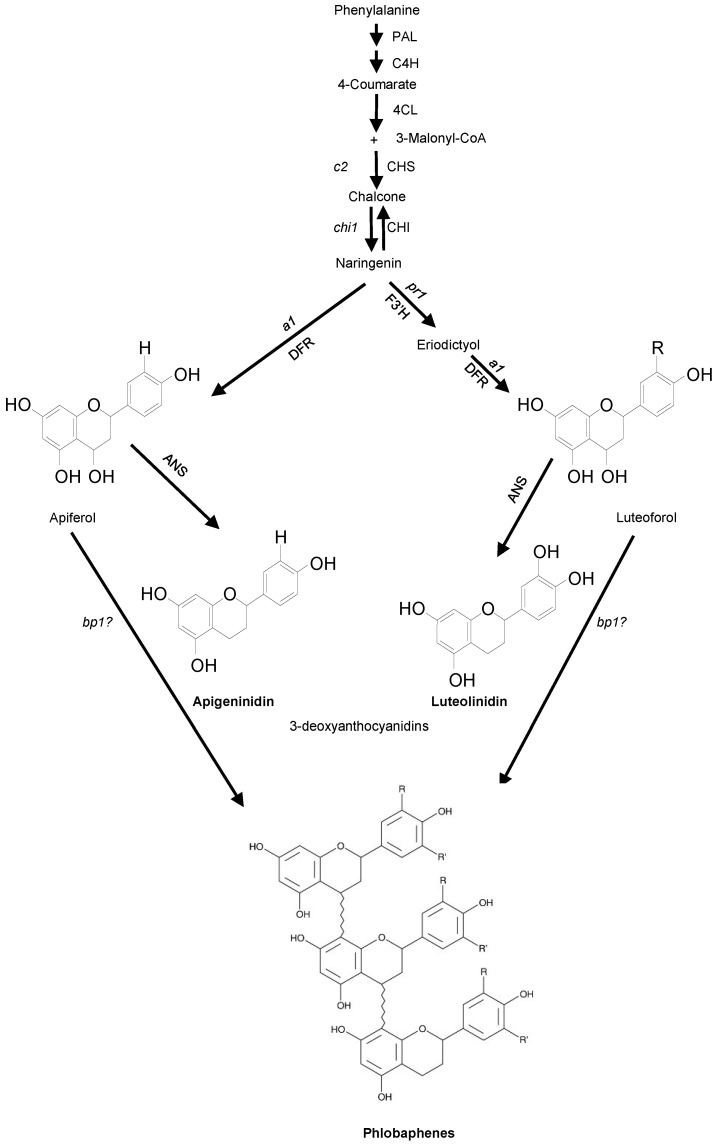
Schematic representation of the biosynthetic pathway of flavonoid compounds. Enzyme names are (gene names in parentheses): PAL, Phenylalanine ammonia lyase; C4H, Cinnamate-4-hydroxylase; 4CL, 4-coumarate: coenzymeA ligase; C3'H, p-coumarate 3'-hydroxlase; CHS (*c2*), Chalcone synthase; CHI (*chi1*), Chalcone isomerase, DFR (*a1*), Dihydroflavonol reductase; and F3'H, Flavonoid 3'-hydroxylase (*pr1*). Pathway modeled after [1,21,22,23,24].

In maize, the flavonoid pathway gives rise to many defense related compounds such as flavan-4-ols, 3-deoxyanthocyanidins, and *C*-glycosyl flavones (Figure 1). Flavan-4-ols are the precursors of the brick red phlobaphene pigments that accumulate in mature pericarp and cob glumes. Their biosynthesis requires a functional *pericarp color1* (*p1*) gene, which encodes an R2R3 MYB transcription factor [6,7,8].

We have performed a comparative characterization of the flavonoid pathway in sorghum and maize [9]. These two species are genetically related and are suggested to have diverged from a common ancestor more than 16.5 million years ago [10,11]. The two genomes have a high degree of synteny and sequence similarity [12,13]. The co-linearity between their genomes may suggest a similarity between their metabolic pathways. In fact, sorghum has also been shown to accumulate phlobaphenes in the pericarp under the control of *yellow seed1* (*y1*), an orthologue of maize *pericarp color1* [14,15,16]. *y1* and *p1* activate the transcription of chalcone synthase (*chs*), chalcone isomerase (*chi*), and dihydroflavonol reductase (*dfr*) during biosynthesis of flavan-4-ols in sorghum and maize [7,9,17].

Regardless of the similarities mentioned above, the flavonoid pathways in sorghum and maize exhibit a number of differences. For example, in maize, phlobaphenes are obvious in the floral tissues, husk, and leaf sheath but not in the leaf, whereas in sorghum these compounds appear in all the above mentioned tissues and in the mature leaf [9]. The presence of phlobaphenes in sorghum leaves may indicate that the *y1* promoter is active in this tissue. Another difference is the response of these two species to fungal challenges. Sorghum responds to anthracnose and other foliar fungi by the induction of red-brown 3-deoxyanthocyanidin phytoalexins [18]. However, there is no published report that leaves of maize lines carrying a similar set of functional flavonoid regulatory and structural genes synthesize detectable levels of 3-deoxyanthocyanidins either constitutively or induced in response to biotic or abiotic stresses. With the exclusion of chalcone synthase in maize, neither the flavonoid structural nor regulatory genes showed induction after fungal infection [19]. Silks of some maize lines have been reported to accumulate very low levels of luteolinidin under the control of *p1* [20].

Sorghum 3-deoxyanthocyanidins include apigeninidin, luteolinidin, and their derivatives. Upon fungal challenge, these compounds accumulate around the primary infection sites and prevent further proliferation of the fungus within sorghum tissues [18,25]. These compounds have been shown to inhibit fungal germ tube growth and distort fungal structures. Their potent antifungal activity against *Colletotrichum sublineolum*, *C*. *graminicola*, and *C*. *heterostrophus* has been demonstrated [26]. 3-deoxyanthocyanidins have a structure similar to flavan-4-ols and their biosynthesis requires the activity of *chs*, *chi*, *dfr*, and *f3'h*. The induction of these genes requires a functional *y1* gene because *y1* mutants are deficient in 3-deoxyanthocyanidins and exhibit symptoms of anthracnose susceptibility [16]. The sequences of *y1* and *p1* genes have a high level of similarity (92%) in the coding region but very poor similarity in the non-coding regions [9]. 

We developed transgenic plants to investigate the heterologous expression of sorghum *y1* in maize and to test if *y1* can induce anthracnose resistance in maize. Our results demonstrate that the *y1* transgenes are active in maize tissues. Biochemical analyses established that *y1* successfully drives the maize–flavonoid pathway towards production of flavan-4-ols and 3-deoxyanthocyanidins. Transgenic *Y1-*maize plants were resistant to both *C. heterostrophus* and *C. graminicola*; this interaction is the result of the induction of 3-deoxyanthocyanidins.

## 2. Results and Discussion

### 2.1. y1 Transgenes Phenocopy p1 Pigmentation Patterns in Maize

**Figure 2:**
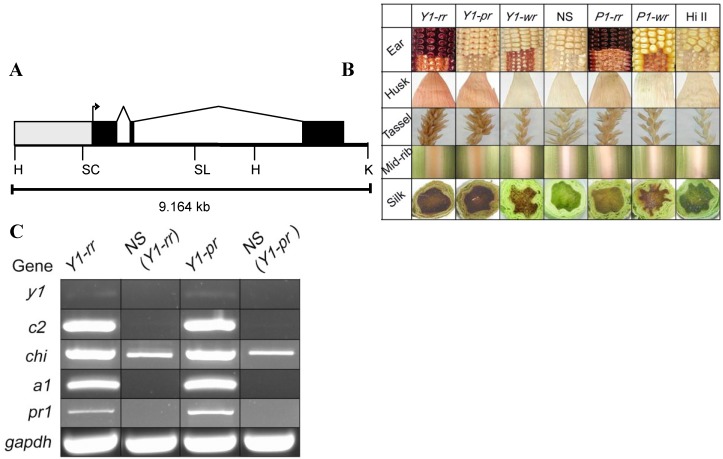
Characterization of *y1* transgenes. (**A**) Structural features of the sorghum *y1* gene. The gray box represents the upstream regulatory region. The bent arrow indicates the transcription start site. Solid boxes correspond to exons that are joined by angled lines representing introns. The restriction enzyme sites shown are: H, *Hin*dIII; K, *Kpn*I; SL, *Sal*I; SC, *Sca*I. Illustration not drawn to scale. (**B**) Sorghum *y1* gene-induced pigmentation phenotypes in transgenic *Y1-*maize. Three *y1* transgenic events representing *Y1-rr*, *Y1-pr* and *Y1-wr* were characterized for ear, husk, tassel glumes, leaf mid-rib, and silk browning phenotypes. Comparable controls included are: plants segregating for the absence of *y1* transgene shown as negative segregant (NS) and native *p1* expressing alleles *P1-rr* and *P1-wr* and *HII* (from A188 X B73), used for transformation. (**C**) Sorghum *y1* gene induces flavonoid structural genes in *Y1-*maize. The expression of the *y1* transgene and four flavonoid structural genes relative to the housekeeping gene glyceraldehyde phosphate dehydrogenase was assayed using RT-PCR. Expression was tested in the pericarp tissues of the *Y1-rr* and *Y1-pr* transgenes and their respective negative segregants (*Y1-rr* and *Y1-pr*). *c2:* chalcone synthase, *chi:* chalcone isomerase, *a1:* dihydroflavonol reductase, *pr1:* flavonoid 3'-hydroxylase, *gapdh:* glyceraldehyde phosphate dehydrogenase.

Transgenic maize lines expressing a sorghum *y1* gene (p*Y1::Y1*) (Figure 2A) exhibited distinct patterns of pericarp and cob glume pigmentation. Three ear pigmentation patterns from independent, representative transformation events and a negative segregant are shown (Figure 2B). The pericarp and cob glume pigmentation patterns described here are based on the nomenclature of the maize *p1* alleles [27]. Transgenic events were divided into four classes based on their ear phenotypes: *Y1-rr* (red pericarp, red cob glumes); *Y1-pr* (patterned pericarp, red cob glumes); *Y1-wr* (white pericarp, red cob glumes); and *y1-ww* (white pericarp, white cob glumes). Sibling maize plants were genotyped using *y1* gene specific primers; those lacking the transgene and showing a susceptible response against BASTA herbicide exhibited a white pericarp and white cob glume phenotype (*y1-ww*). These negative segregants (NS) represented similar genetic background to *Y1* transgenic events and thus were used as controls throughout this study.

Unlike the maize *p1* gene, the sorghum *y1* gene induced accumulation of phlobaphenes in the husk and tassel glumes of the three functional categories of transgenic events (*Y1-*maize). Apart from the accumulation of phlobaphenes in floral tissues, an orange pigment was also observed in the leaf midrib in *Y1*-maize. The mid-rib pigmentation appeared at the three-leaf stage of plant growth and persisted through the maturation of the plant. Additionally, the silk tissue of *Y1*-maize plants showed a rapid “silk-browning” phenotype at the cut ends or upon injury. The silk browning phenotype is thus under the control of the *y1* transgene and is similar to the one produced by *p1* and *p2* genes in maize [28]. In *Y1-*maize, this phenotype is more intense compared to the one observed with the endogenous *p1* alleles (see Figure 2B). These distinct phenotypes produced by *Y1*-maize were stably inherited across seven generations. To further confirm if *y1* regulated phenotypes are the result of the activation of flavonoid structural genes in transgenic maize, expression of the *y1* transcription factor and four marker genes was assayed: chalcone synthase (*c2*), chalcone isomerase (*chi*) dihydroflavonol reductase (*a1*), and flavonoid 3'-hydroxylase (*pr1*). Pericarp tissues of the *Y1-rr* and *Y1-pr* transgenic events showed induction of *c2*, *a1*, and *pr1*, and upregulation of *chi* flavonoid structural genes and the *y1* transcription factor in *Y1-*maize while tissue obtained from their respective NS plants showed no detectable expression by RT-PCR (Figure 2C). Overall our phenotypic and gene expression data demonstrated that the sorghum *y1* gene can target maize flavonoid structural genes and either induce or upregulate the flavonoid biosynthetic pathway in maize floral and vegetative tissues.

### 2.2. y1 Regulates Accumulation of 3-Deoxyflavonoids (flavan-4-ols) in Maize

**Figure 3:**
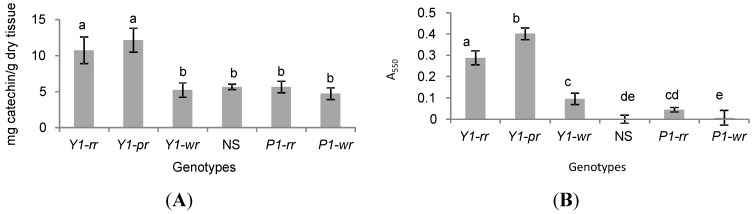
Sorghum *y1* gene induces accumulation of flavonoid compounds in transgenic maize leaves. *Y1-rr*, *Y1-pr*, and *Y1-wr* are independent transgenic events; NS, Negative segregant; *P1-rr* and *P1-wr*, maize lines carrying endogenous *p1* alleles. Values shown are mean ± SE. (**A**) Total flavonoids expressed as catechin equivalents; (**B**) Flavan-4-ols expressed as absorbance at 550 nm.

In sorghum, *y1* has been shown to be required for the biosynthesis of flavan-4-ols or 3-deoxyflavonoid compounds that are precursors to the phlobaphenes [9]. To investigate the effect of *y1* on the flavonoid pathway in maize, we assayed flavan-4-ol accumulation in the pericarp, cob glumes, silks, and leaves (see Figure S1). Spectral results indicated the presence of flavan-4-ols with an absorption maximum of 564 nm. Quantitative measurement of total flavonoids in the leaf showed significantly higher accumulation in *Y1-rr* and *Y1-pr* as compared to *Y1-wr* (*p* = 0.0039 and *p* = 0.0152, respectively) and the endogenous *p1* alleles (*p* ≤ 0.01, Figure 3A). The high level of flavonoid compounds in the two *y1* transgenes could be due to the accumulation of flavonoid pathway intermediates such as chalcone and naringenin or novel compounds produced by the activity of maize enzymes induced by an active *y1*.

To further identify compounds regulated by the *y1* gene, we surveyed the leaves of *Y1-*maize plants for the presence of flavonoid precursors that give rise to either phlobaphenes or anthocyanins. The acid-butanol extracts were boiled to differentiate between flavan-4-ols and flavan-3,4-diols. In maize, flavan-4-ols (3-deoxyflavonoids) give rise to flavylium cations that have a λ_max_ of 564 nm and are heat labile, while flavylium cations obtained from the flavan-3,4-diol (3-hydroxyflavonoids)-derived compounds exhibit a λ_max_ of 533 nm and are unaffected by boiling [21,29]. Our results revealed that the *Y1*-maize extracts exhibited a major peak at 564 nm, which disappeared upon boiling, confirming the presence of flavan-4-ols (See Supplemental Figure S1). Quantification of flavan-4-ols in leaf tissue revealed that *Y1-rr* and *Y1-pr* transgenic events had significantly higher levels of these compounds as compared to the endogenous *p1* alleles and NS (*p* < 0.0001). Although *Y1-wr* transgenics accumulated significant levels of flavan-4-ols compared to the NS and *P1-wr* [B73] (*p* = 0.0178 and *p* = 0.0008 respectively), they did not significantly differ from the *P1-rr* allele (*p* = 0.1692) in this trait (Figure 3B).

**Figure 4:**
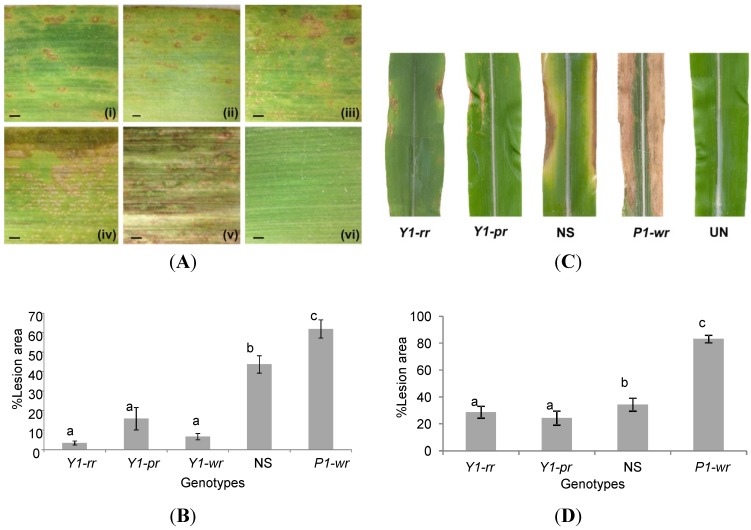
Sorghum *y1* gene enhances resistance against *C. heterostrophus* and *C. graminicola* in *Y1-*maize. (**A**) Detached leaf assay showing disease symptoms that developed 4 days post infection (dpi) when infected with *C. heterostrophus*. (**i**) *Y1-rr*; (**ii**) *Y1-pr*; (**iii**) *Y1-wr*; (**iv**) NS; (**v**) *P1-wr*; (**vi**) un-inoculated *Y1-pr*. Scale bar indicates 1 mm. (**B**) Quantification of the lesion area 4 dpi with *C. heterostrophus*. Values shown are the mean ± SE. (**C**) Symptoms that developed 11 dpi when whole plants were infected with *C. graminicola*. (**D**) Quantification of lesion area 11 dpi with *C. graminicola*. Values shown are the mean of 44 replicates ± SE. The x-axis in Figure 4A,C shows different genotypes used: *Y1-rr*, *Y1-pr*, *Y1-wr*; NS and *P1-wr*.

### 2.3. Y1-Maize Exhibits Enhanced Resistance to C. heterostrophus and C. graminicola 

In sorghum, a functional *y1* gene is required for resistance against *C. sublineolum* [16]*.* To test the response of *Y1-*maize to fungal challenges, plants were infected with either *C. heterostrophus* or *C. graminicola*. We compared the response of *Y1*-maize plants to both NS and *P1-wr*. When infected with *C. heterostrophus*, the inoculated leaves of *Y1-*maize plants produced a reduced number of chlorotic lesions compared to the control genotypes (Figure 4A). In different *Y1-* maize events, the mean values of the infected area ranged from 4% to 16% (Figure 4B). In contrast, these lesions were spread over about 40% and 62% of the leaf area in NS and *P1-wr* genotypes, respectively. These results indicate that the disease severity was significantly reduced in *Y1-*maize plants compared to the control genotypes (*p* < 0.01). Similarly, when entire plants were infected with *C. graminicola*, we found that the two transgenic maize lines (*Y1-rr* and *Y1-pr*) were more resistant, with averages of only 24%–29% of the leaf covered in lesions compared to the NS and *P1-wr*, which had 34% and 83% lesion area, respectively (*p* < 0.05) (Figure 4C,D).

### 2.4. Induction of 3-Deoxyanthocyanidins during Y1-Maize–C. graminicola Interaction

**Figure 5:**
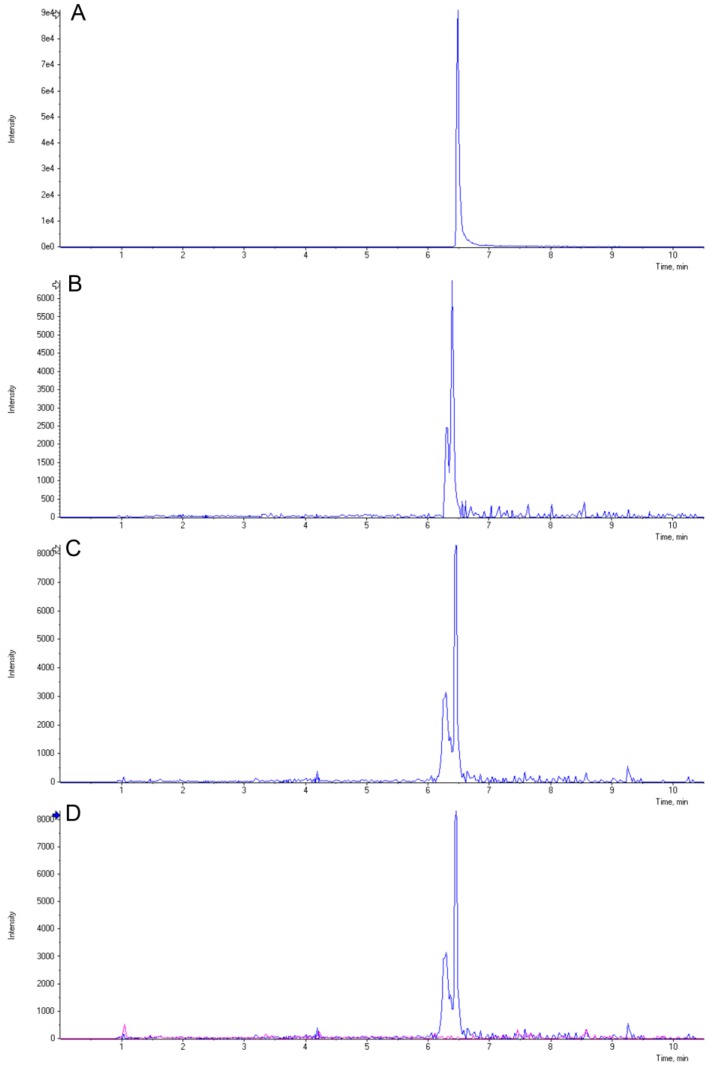
Induction of 3-deoxyanthocyanidins and their derivatives in *Y1-*maize. LC-MS chromatograms obtained from the luteolinidin standard (**A**), infected leaves of *Y1-rr* ((**B**,**C**) representing two biological replicates), and an overlay (**D**) of *Y1-rr* (sample B; blue trace) and NS (pink trace) are presented for comparison. The *m*/*z* values of the extracted chromatograms were similar to those of the luteolinidin standard (271.060).

Transgenic maize plants carrying the sorghum *Y1* gene were shown to be more resistant to the foliar pathogens *C. graminicola* and *C. heterostrophus* relative to the NS*.* In sorghum, resistance to foliar pathogens is in part due to the induced biosynthesis of 3-deoxyanthocyanidins [16]. Similarly, the resistant phenotype relative to the NS may be due to the biosynthesis of these novel compounds driven by the *Y1* gene in maize. Extracts obtained from infected leaves were analyzed using LC-MS to identify these compounds. The *m/z* ratios and elution times of peaks similar to those of apigeninidin and luteolinidin were scrutinized. Chromatograms obtained from the *Y1* transgenes *Y1-rr* and *Y1-pr* indicated novel peaks eluting with a retention time and *m/z* ratio similar to luteolinidin (271.060) compared to the NS (Figure 5), though no peaks were indicative of apigeninidin. 

### 2.5. Discussion

In the current study, the activity of the sorghum *y1* gene was tested as a transgene in maize. First, our results established that, similar to P1, the Y1 protein is able to activate the same suite of known maize flavonoid genes, resulting in maize-like phlobaphene accumulation patterns in seed pericarp and cob glumes. In addition, the *y1* gene also induced the biosynthesis of flavan-4-ols in maize leaves, a property that has not been reported for maize lines expressing *P1-rr* or *P1-wr* [27,30]. The presence of flavan-4-ols in these leaves suggests that *y1* behaves in maize as it does in sorghum and actively interacts with the promoters of flavonoid genes to drive the pathway towards the production of these flavonoid compounds in the maize leaf. In *Y1-*maize, we observed phlobaphenes in the mature leaf tissue, which suggests that, like sorghum, the polymerization of flavan-4-ols to phlobaphenes can occur as also documented in the case of a maize mutant *Unstable factor for orange1* (*Ufo1*) [31,32]. Thus, the absence of flavan-4-ols and phlobaphenes in maize leaves containing active endogenous *p1* alleles could be due to poor activity of the *p1* or the inability of P1 to activate transcription of flavonoid structural genes in leaves [27,28,30]. The biochemical analysis of the *Y1-*maize leaves revealed that Y1 induced significant accumulation of flavan-4-ols and flavonoids to levels that are not commonly found in maize lines. This further establishes that the *y1* promoter is active in maize leaves.

The *Y1-*maize plants showed enhanced resistance against *C. graminicola* and *C. heterostrophus*. LC-MS profiling of induced flavonoids showed the presence of 3-deoxyanthocyanidin phytoalexins, specifically luteolinidin. In addition to luteolinidin, a second unknown small peak is observed in *Y1-*maize, which is absent from the NS profile. The improved disease resistance of *Y1-*maize plants is thus due to the induced 3-deoxyanthocyanidins as well as higher levels of pre-formed flavonoids, especially flavan-4-ols, which are known to contribute to plant defense [33]. Flavan-4-ols have also been suggested as putative precursors of 3-deoxyanthocyanidins [6,17,21,34,35,36,37]. Sorghum grains and leaves with higher levels of flavan-4-ols exhibited better resistance against mold compared to those that were deficient [38,39,40]. Flavan-4-ols include two main compounds—luteoforol and apiforol. Luteoforol has been demonstrated to have potent biocidal effects against many fungi and bacteria, including *C. graminicola* [41]. This antimicrobial activity might justify its presence in the epidermal cells of pericarp, silk, husk, and leaves. In fact, a mechanism describing the release of flavan-4-ols from their intracellular compartments to the sites of pathogen infection, similar to that of sorghum 3-deoxyanthocyanidins, has been proposed [18,25,41]. Although we were able to identify luteolinidin, we did not detect any apigeninidin, possibly due to a very active flavonoid 3′-hydroxylase, which converts apigeninidin to luteolinidin [23,42].

Induction of phenylpropanoids in maize cell suspensions after transformation with the *p1* transgene has been reported [21,24,43]. These, along with our current results, demonstrate that *p1* and *y1* transgenes play similar regulatory roles in the phenylpropanoid pathway in maize. However, the basis of this mechanism is not yet clear. One possibility is that the R2R3 MYB protein products of *p1* and *y1* genes might interact directly with structural genes in the phenylpropanoid pathway to secure the efficient flow of intermediates between the phenylpropanoids and flavonoids. In fact, in BMS maize cells, the *p1* transgene induced the expression of *pal1*, which controls the flow of the amino acid phenylalanine into the phenylpropanoid pathway [43]. Since *y1* and *p1* are known to regulate flavonoid biosynthesis downstream of chalcone, it is also possible that these transcription factors may interfere with feedback regulation controlling the activity of enzymes working in other branches of the phenylpropanoid pathway [21,32,44,45,46]. Thus future exploitation of *y1*, an R2R3 MYB regulatory gene, to produce desirable biopesticides is a viable strategy [2,47,48,49,50].

## 3. Experimental Section

### 3.1. Maize Genetic Stocks

Maize genetic stocks of inbred lines 4co 63 (*p1-ww*) and B73 (*P1-wr*) were obtained from Maize Genetic Coop Center, USDA, Urbana-Champaign, IL, USA. Genetics stocks carrying *p1* alleles *P1-rr 4B2*, *P1-ww-1112* and *p-del2* were obtained from Thomas Peterson, Iowa State University, Ames, IA, USA.

### 3.2. Transgene Constructs

All plasmids used in this study were developed based on the pBluescript II vector (Stratagene, La Jolla, CA, USA). The plasmid p*Y1::Y1* contains 9164 bp of the *y1* gene [AY860968 [16]], which includes the 2375 bp of the 5' regulatory region, 6946 bp sequence with three exons and two introns, and 820 bp of the 3′UTR. This plasmid was prepared by ligating the *Hin*dIII-*Kpn*I DNA fragment of the *Y1-rr* gene into a pBluescript II vector.

### 3.3. Tissue Culture, Transformation, and Regeneration of Transgenic Maize Plants

We used maize *HiII* line as a transgene recipient because it carries a non-functional *p1* allele. *HiII* was developed from a cross between A188 × B73 [51]. Immature zygotic embryos derived from the *HiII* maize [52] were used to develop friable embryogenic type II calli. Callus induction, maintenance, and transformation were carried out according to a previously described protocol at the Plant Transformation Facility at Iowa State University [53]. A plasmid carrying the *BAR* gene for Bialphos herbicide tolerance was co-bombarded, along with the p*Y1:Y1* construct. A total of 16 p*Y1:Y1* independent transformation events were generated from calli resistant to the herbicide Bialphos (BASTA™, AgrEvo, Wilimington, DE, USA). The selection for transgenic plants in T_1_ and subsequent generations was based on herbicide resistance as well as PCR analysis, using *y1* specific gene primers. The transgenic plants were maintained in a hemizygous state by out-crossing with pollen from the inbred line 4Co63 that carries a *p1-ww* allele (null *p1* allele). Progenies derived from such crosses always segregated in a 1:1 ratio, indicating stable expression patterns of the transgenic plants included in this study. All maize plants carrying p*Y1::Y1* transgenes exhibited normal growth and morphology when compared with the sibling negative segregant (NS) maize plants.

### 3.4. Expression Analysis of Genes Induced by y1

Total RNA was extracted from pericarp 18 days after pollination (dap) by RNAzolRT (Molecular Research Center Inc., Cincinnati, OH, USA) and used to synthesize first strand cDNA using the High-Capacity cDNA Reverse Transcription Kit (Applied Biosystems, Foster City, CA, USA). PCR using primers specific to flavonoid pathway genes was used to determine the expression of said genes: yellow seed 1 (*y1*): RT_PWREx_2F (5'-TCCGGTGCGGCAAGAG-3') and RT_PWREx_2R (5'-GGAGCTTGATGATGATGTCTTCTTC-3'); chalcone synthase (*c2*): CHSF (5'-TCGATCGGTCTCTCTGGTACAACGTA-3') and CHSR (5'-TACATCATGAGGCGGTTCACGGA-3'); chalcone isomerase (*chi1*): CHIF (5'-GTGCGGAATTTAACATGGCGTGC-3') and CHIR (5'-CGGCGCGAAAGTCTCTGGCTT-3'); flavonoid 3'-hydroxylase (*pr1*): 5F3H-F2 (5'-GAGCACGTGGCGTACAACTA-3') and ZMR4 (5'-AAACGTCTCCTTGATCACCGC-3'); dihydroflavonol reductase (*a1*): A1 (5'-CAATTCGTTGAACATGGAAGTAAG-3') and A2 (5'-CAATTCGTTGAACATGGAAGTAAG-3') and glyceraldehyde 3-phosphate dehydrogenase (*gapdh*): GAP1 (5'-AGGGTGGTGCCAAGAAGGTTG-3') and GAP2 (5'-GTAGCCCCACTCGTTGTCGTA-3').

### 3.5. Tissue Collection for Chemical Analyses

All tissues used for chemical analyses were collected at the specified time, flash frozen in liquid nitrogen and either lyophilized or stored at −80 °C. All analyses were performed on three independent sample replicates.

### 3.6. Quantification of Total Flavonoids and Flavan-4-ols

All biochemical analyses were carried out on the second leaf above the primary ear of greenhouse grown plants collected at the time of pollination. For total flavonoid quantification, ground tissue (20 mg) was washed three times in ether to remove waxes and chlorophyll pigments and then extracted three times under sonication in 70% acetone supplemented with 1 mM ascorbic acid. The supernatant was collected and acetone was evaporated using a speed vacuum drier. The extract was used for determination of total flavonoids [54]. The extracts were diluted with 1 M NaOH and the absorbance was recorded at 510 nm using a SpectraMAX 190 plate reader (Molecular Devices Corp., Sunnyvale, CA, USA). Total flavonoid content was expressed as mg catechin equivalent g^−1^ dry weight.

To quantify flavan-4-ols, 30 mg ground leaf tissue was washed in ether and suspended in 500 µL of HCl:butanol (3:7). The homogenate was incubated at 37 °C for 1 h, followed by centrifugation at 20,000 *g* for 10 min. The absorbance of the supernatant was recorded at 550 nm using an UV mini-1240 spectrophotometer (Shimadzu Scientific Instruments, Inc. Columbia, MD, USA). The flavan-4-ols were expressed as the relative concentration of flavylium ions [21].

### 3.7. Evaluation of Y1-Transgenic Plants for Resistance to Cochliobolus heterostrophus and Colletotrichum graminicola

Resistance to southern corn leaf blight caused by *C. heterostrophus* was evaluated using the detached leaf assay [55]. *C. heterostrophus* was grown on potato dextrose agar (PDA) under continuous light at room temperature for ten days. Conidia were collected in 0.001% Tween 20. The second leaf above the primary ear was collected 15 days after pollination (dap). Leaf discs were prepared from both sides of the midrib and those on the left side were used as controls. Discs with adaxial surface facing upward were placed on water agar (1% w/v) supplemented with 2 mg·L^−1^ kinetin. Whatman filter papers soaked in either a spore suspension of *C. heterostrophus* (10^5^ spores·mL^−1^) or 0.001% Tween 20 for the control were placed on the leaf discs. Plates were incubated under illumination at 28 °C. The filter paper discs were removed after 24 h and plates were kept under the same conditions until collection. The disease phenotypes of the p*Y1:Y1* transgenic plants and control genotypes were recorded using a dissection microscope (Nikon SMZ1000) connected to a Nikon digital camera (DXM1200F). Disease severity was quantified as described below.

To test the role of *y1* in resistance to anthracnose leaf blight, four- to six-week-old greenhouse-grown plants were inoculated and disease was quantified using *C. graminicola* as described previously [16]*.*

### 3.8. Image Analysis for Evaluation of Disease Response

For quantitative analysis, images were processed by Automated Lesion Extraction using algorithms (PhenoPhyte) developed for a visual phenotype database [42]. This technique depends on differentiating the lesion pixels (foreground) from the healthy ones (background) and measuring the area of lesions. The percentage of the infected area was used to evaluate disease severity.

### 3.9. LC-MS Analysis of 3-Deoxyanthocyanidins

Flag leaves of field-grown plants were used to identify compounds induced in response to *C. graminicola* infection, using the detached leaf assay as described above and harvested for analysis 3 dpi. Infections were carried out in triplicate for each genotype, each of which consisted of three individual leaves. Tissue samples (~100 mg) were extracted in 2 mL of 2 N HCl by boiling for 40 min, then centrifuged at 20,000 *g* for 15 min. The resulting supernatant was extracted twice in 1 mL of isoamyl alcohol, which was evaporated to dryness and re-suspended in 250 µL of methanol supplemented with 0.1% HCl [56]. Extracts (5 µL) were separated by reverse phase HPLC using a Prominence 20 UFLCXR system (Shimadzu, Columbia, MD, USA) with a Waters BEH C18 column (100 × 2.1 mm, 1.7 µm particle size) and a 20 min aqueous/acetonitrile gradient, at a flow rate of 250 µL/min. Solvent A was water with 0.1% formic acid, and Solvent B was acetonitrile with 0.1% formic acid. The initial conditions were 97% A and 3% B, increasing to 45% B at 10 min and 75% B at 12 min, then held at 75% B until 17.5 min before returning to the initial conditions at 18 min. The eluate was delivered into the 5600 (QTOF) TripleTOF using a Duospray™ ion source (all AB Sciex, Framingham, MA, USA), samples were analyzed in positive ion mode, and the mass spectrometer was operated in IDA (Information Dependent Acquisition) mode with a 100 m survey scan from 50 to 1250 *m*/*z*, and up to 10 MS/MS product ion scans per duty cycle. The survey scan data was used to generate the extracted ion chromatographs with PeakView software package (AB Sciex, Framingham, MA, USA).

## 4. Conclusions

We engineered 3-deoxyanthocyanidin phytoalexins in maize by transforming it with a sorghum transcription factor, *yellow seed 1* (*y1*). In maize, *Y1* expression drives the biosynthesis of foliar flavonoid compounds, especially flavan-4-ols. Furthermore, fungal infection resulted in the induction of luteolinidin, which is a potent antifungal compound. We believe the preformed flavan-4-ols, in addition to the induced luteolinidin, contributed to increased resistance of the transgenic maize compared to the near-isogenic non-transgenic lines. The introduction of *y1* is thus a viable strategy for introducing anthracnose resistance to maize lines carrying the downstream flavonoid pathway structural genes.

## Supplementary Materials

Supplementary materials can be accessed at: http://www.mdpi.com/1420-3049/20/02/2388/s1.
