# Supplementary material for: A Sorghum MYB Transcription Factor Induces 3-Deoxyanthocyanidins and Enhances Resistance against Leaf Blights in Maize
Source: Molecules. 2015 Jan 30;20(2):2388–404. doi: 10.3390/molecules20022388 (PMC6272393; doi:10.3390/molecules20022388)
Supplement: Supplementary file 1 [file molecules-20-02388-s001.pdf]

Supplementary Materials

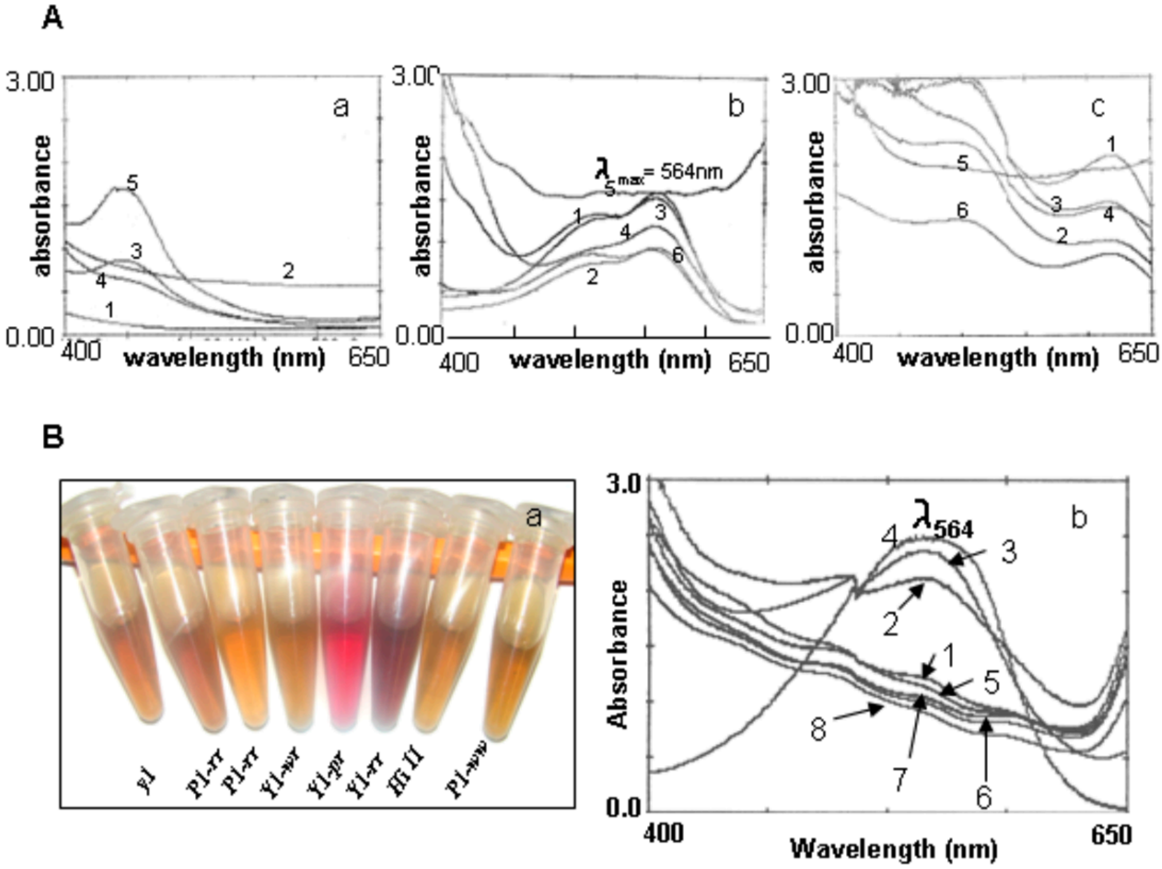

Figure S1. Spectral analysis of flavonoids from floral and vegetative tissues of transgenic and non-transgenic maize plants. (**A**) Spectral analysis of acidic-butanol extracts from sibling maize plants segregating for either the absence (a) or the presence (b) of *y1* transgene. The absorption maxima at 564 nm were eliminated by boiling confirming the presence of flavan-4-ols (c). Numbers on curves represent extracts prepared from: pericarp (1), cob glumes (2), tassel glumes (3), anthers (4), and mid-rib (5). *P1-rr* Pericarp extract (curve 6) was used as a positive control for flavan-4-ols. (**B**) Spectral analysis of acidic-butanol extracts from mature leaves of transgenic and non transgenic maize plants. Extracts from leaves of standard maize lines expressing native *P1-rr* and *P1-wr* alleles were included for comparison. Leaves were deprived of chlorophyll followed by acidic-butanol assay. The clear extracts were photographed (a) and screened for the presence of flavan-4-ols (b). Curves are for leaf extracts from: 1, *Y1-wr*; 2, *Y1-rr*; 3, *Y1-pr*; 4, *P1-rr* pericarp (positive control for flavan-4-ols); 5, NS; 6, HiII; 7, *P1-rr*; and 8, *P1-wr*.
